# Primary Non-Aortic Lesions Are Not Rare in Marfan Syndrome and Are Associated with Aortic Dissection Independently of Age

**DOI:** 10.3390/jcm12082902

**Published:** 2023-04-17

**Authors:** Jean Sénémaud, Marine Gaudry, Elisabeth Jouve, Arnaud Blanchard, Olivier Milleron, Yves Dulac, Laurence Olivier-Faivre, Dominique Stephan, Sylvie Odent, Damien Lanéelle, Sophie Dupuis-Girod, Guillaume Jondeau, Laurence Bal-Theoleyre

**Affiliations:** 1 Service de Chirurgie Vasculaire, Thoracique et de Transplantation Pulmonaire, AP-HP, CHU Bichat, 75018 Paris, France; 2 Service de Chirurgie Vasculaire, AP-HM, CHU La Timone, 13385 Marseille, France; marine.gaudry@ap-hm.fr; 3 Service d’Evaluation Médicale, AP-HM, CHU La Conception, 13005 Marseille, France; elisabeth.jouve@ap-hm.fr; 4 Centre de Référence Constitutif Pour le Syndrome de Marfan et Apparentés, Centre Aorte Timone, AP-HM, CHU La Timone Adultes, 13014 Marseille, France; arnaud.blanchard@ap-hm.fr (A.B.); laurence.bal@ap-hm.fr (L.B.-T.); 5 Centre National de Référence Pour le Syndrome de Marfan et Apparentés, VASCERN HTAD European Reference Centre, Service de Cardiologie, AP-HP, CHU Bichat, INSERM U 1148 LVTS, Université de Paris, 75014 Paris, France; olivier.milleron@aphp.fr (O.M.); guillaume.jondeau@aphp.fr (G.J.); 6 Centre de Référence Constitutif Pour le Syndrome de Marfan et Apparentés, Hôpital des Enfants, CHU de Toulouse, 31300 Toulouse, France; dulac.y@chu-toulouse.fr; 7 Centre de Compétence Pour le Syndrome de Marfan et Apparentés, Hôpital des Enfants, CHU de Dijon, 21000 Dijon, France; laurence.faivre@chu-dijon.fr; 8 Centre de Compétence Pour le Syndrome de Marfan et Apparentés, CHU Nouvel Hôpital Civil, 67000 Strasbourg, France; dominique.stephan@chru-strasbourg.fr; 9 Centre de Compétence Pour le Syndrome de Marfan et Apparentés, Hôpital Sud, CHU de Rennes, 35200 Rennes, France; sylvie.odent@chu-rennes.fr; 10 Centre de Compétence Pour le Syndrome de Marfan et Apparentés, CHU de la Côte de Nacre, 14033 Caen, France; med-vasculaire-sec@chu-caen.fr; 11 Centre de Compétence Pour le Syndrome de Marfan et Apparentés, CHU Hôpital Louis Pradel, 69500 Lyon, France; sophie.dupuis-girod@chu-lyon.fr

**Keywords:** Marfan syndrome, non-aortic aneurysm, ectasia, aortic dissection

## Abstract

Purpose: The study sought to estimate the prevalence of primary non-aortic lesions (PNAL) unrelated to extension of aortic dissection (AD) in a cohort of patients with Marfan syndrome (MFS). Methods: Adult patients presenting with pathogenic FBN1 mutations and an available pan-aortic contrast-enhanced CTA in eight French MFS clinics from April to October 2018 were included. Clinical and radiological data, particularly the presence of aortic lesions and PNAL (including aneurysm and ectasia), were retrospectively analyzed. Results: Out of 138 patients, 28 (20.3%) had PNAL. In total, 27 aneurysms in 13 patients and 41 ectasias in 19 patients were reported mainly in the subclavian, iliac, and vertebral segments. Four patients (31%) with aneurysms and none with ectasia required prophylactic intervention during follow-up (median: 46 months). In multivariate analysis, factors associated with PNAL were history of AD (OR = 3.9, 95%CI: 1.3–12.1, *p* = 0.018), history of previous descending aortic surgery (OR = 10.3, 95%CI: 2.2–48.3, *p* = 0.003) and age (per 10 years OR = 1.6, 95%CI: 1.1–2.4, *p* = 0.008). Conclusion: PNAL is not rare in MFS patients with evolutive aortic disease. Natural history may differ between aneurysms and ectasia, emphasizing the need for standardized definitions and systematic screening for PNAL.

## 1. Introduction

Marfan syndrome (MFS) is an autosomal dominant multiorgan disease usually related to FBN1 gene pathogenic variants [1]. The extent and progression of organ lesions are variable [2], but life expectancy remains strongly correlated with aortic disease [3,4]. Extra-aortic aneurysms could be expected in MFS because of the widespread nature of fibrillin 1, but the presence of non-aortic lesions (NAL) is not considered as a diagnostic criterion in MFS [5]. However, the recent 2019 ESVS guidelines recommend a complete overview of cerebral, thoracic, and abdominal vasculature in MFS patients [6]. Indeed, a growing body of evidence underlines the importance of NAL; Yetman et al. noted that one-third of the adults in their MFS cohort presented with non-aortic aneurysms, which were incidentally discovered during follow-up imaging [7], and Schoenhoff et al. reported a 20% rate of non-aortic intervention during follow-up of a large MFS cohort [8]. Little is known about the prevalence and prognosis of non-aortic aneurysms. Most studies that report their presence in MFS patients lack the precision to distinguish primary NAL (not ensuing from a previous aortic dissection (AD)) from secondary NAL related to the extension of an AD associated with aneurysmal degeneration. Moreover, the clinical significance of these lesions remains unclear [9], although a recent study associated extra-aortic aneurysms with a greater risk for aortic surgery in MFS [10].

The focus of this paper is to describe the extra-aortic arterial disease in patients with FBN1 gene mutation, and to provide insights into their clinical consequences. We thus aimed to assess the prevalence and distribution of primary non-aortic lesions (PNAL) and to identify the factors associated with PNAL in a multicenter cohort of MFS patients with FBN1 mutation.

## 2. Materials and Methods

### 2.1. Study Population and Design

Adult patients presenting with pathogenic FBN1 mutations with at least one pan-aortic contrast-enhanced computed tomographic angiogram (CTA) in eight French MFS clinics from April to October 2018 were retrospectively included. Molecular diagnostics of all patients included in this study were performed at the Department of Genetics (Pr. Boileau, Assistance Publique-Hopitaux de Paris) using standard procedures as previously reported [10]. All FBN1 mutations were validated using Sanger sequencing. Demographic features (sex, age, weight, height, smoking status, presence of hypertension, coronary artery disease, chronic kidney disease, history of cardiac surgery and/or AD, and history of thoracic aortic surgery), radiological findings at baseline, and clinical follow-up (FU after index CTA) data were recorded in a dedicated database. Index CTAs represented the patients’ baselines and allowed (1) the comparison of the occurrence of AD in PNAL and non-PNAL patients from birth to the baseline; (2) statistical analysis to identify factors associated with the presence of PNAL (given that PNAL could be only assessed using the index CTAs); and (3) a clinical follow-up from the index CTA to the last FU recorded, enabling analysis of the outcomes of PNAL. Missing and FU data were obtained from medical records and correspondence with the patient or referring practitioner.

### 2.2. Ethical Considerations

This study complies with the Declaration of Helsinki and was approved by the legal entities (Commission Nationale de l’Informatique et des Libertés) and the Ethics Committee (Comité de Protection des Personnes—CPP) required by French regulations. Institutional review board (IRB) approval was obtained from the CPP Ile de France XI (registration number 11008). All patients gave signed informed consent.

### 2.3. Imaging

All patients underwent one pan-aortic contrast-enhanced CTA (1 mm slices), including the cervical, thoracic, and abdominal arterial branches, allowing the assessment of peripheral arterial vasculature from the supra-aortic trunks to the common femoral segments. Non-aortic segments included the innominate artery, carotid arteries, vertebral arteries, subclavian and axillary arteries, coeliac artery, superior and inferior mesenteric arteries, splenic and hepatic arteries, renal arteries, iliac arteries, and common femoral segments. Intracranial vasculatures along upper and lower limb segments were not assessed. In our group, such CTAs were performed in the following cases: enlargement of the aortic root using ultrasonography during usual follow-up >3 mm/year, preoperative evaluation before elective cardiac/aortic surgery and after the occurrence of an AD. Measurements included aortic (aortic root, tubular aorta and arch, descending aortic, and thoracoabdominal aortic segments) and non-aortic segments using centerline reconstructions (3Mensio workstation; Pie Medical Imaging, Bilthoven, The Netherlands). All CTA images were reviewed independently by two blinded senior surgeons (JS and MG), and discrepancies were resolved by consensus. All patients underwent annual echocardiography in the referral MFS clinic. Surveillance of PNAL was left at the physicians’ discretion.

### 2.4. Outcomes

AD was reported using the Stanford classification [4]. Non-aortic lesions were systematically evaluated for each patient. An artery was considered dilated when the arterial diameter was above predefined values described by Schoenhoff et al.: common carotid artery ≥10 mm for men and ≥9 mm for women, subclavian/axillary artery ≥11 mm, iliac artery ≥16 mm for men and ≥13 mm for women, and femoral artery ≥13 mm for men and ≥10 mm for women [8]. Localized non-aortic dilations, including loss of parallelism of the arterial wall associated with maximal diameters exceeding 150% of those of the adjacent segments, were classified as aneurysms [6,11]. Conversely, non-aortic dilations presenting with integrity of arterial wall parallelism and a dilation <150% of the expected arterial diameter were classified as ectasia [12]. Primary NAL included peripheral arterial aneurysms or ectasia “spontaneously” present in the arterial branches and not ensuing from an aortic or arterial dissection. Secondary NAL included dissected arterial segments with aneurysmal evolution associated with an AD and/or peripheral arterial lesions related to previous extracorporeal circulation cannulation sites. Such secondary non-aortic lesions were excluded from analysis.

### 2.5. Clinical Follow-Up

The clinical follow-up (FU) was defined as the period between the time of analyzed CTA (baseline) and the last available clinical evaluation for each patient. All patients underwent annual follow-up at their respective MFS clinics. The standard FU included a clinical examination and echocardiography. PNAL follow-up was performed using either ultrasound or CTA imaging and was left to the discretion of the referent physician.

### 2.6. Statistical Analysis

Analyses were performed using the Statistical Package for the Social Sciences software, version 20 (SPSS; IBM Corporation, Armonk, NY, USA). Continuous variables are described using medians and interquartile ranges (IQRs). Categorical variables are presented as numbers and frequencies. Univariate analyses between the two groups of patients, PNAL and non-PNAL, were performed using the Mann–Whitney test for continuous variables (age at CTA) and Chi-square or Fisher’s exact test for categorical variables (gender, history of cardiac surgery or AD or previous descending aortic surgery). All variables having a *p* value < 0.25 in univariate testing or with previously demonstrated relationships were implemented in a logistic model for multivariate analysis. The multivariate model was summarized by the odds ratio (OR) and a 95% confidence interval (95% CI). The Hosmer–Lemeshow test was applied to assess the goodness of fit of the logistic regression model.

Dissection-free survival was estimated using the Kaplan–Meier method and compared by log-rank testing between the two groups. Curves began at the patients’ birth dates, and censoring was applied at the dates of AD occurrence or at the dates the CTA was performed (right censoring). Hazard ratios (HR) were calculated with 95% CI. All tests were two-tailed, and statistical significance was defined as *p* < 0.05.

## 3. Results

### 3.1. Cohort Description

**Figure 1:**
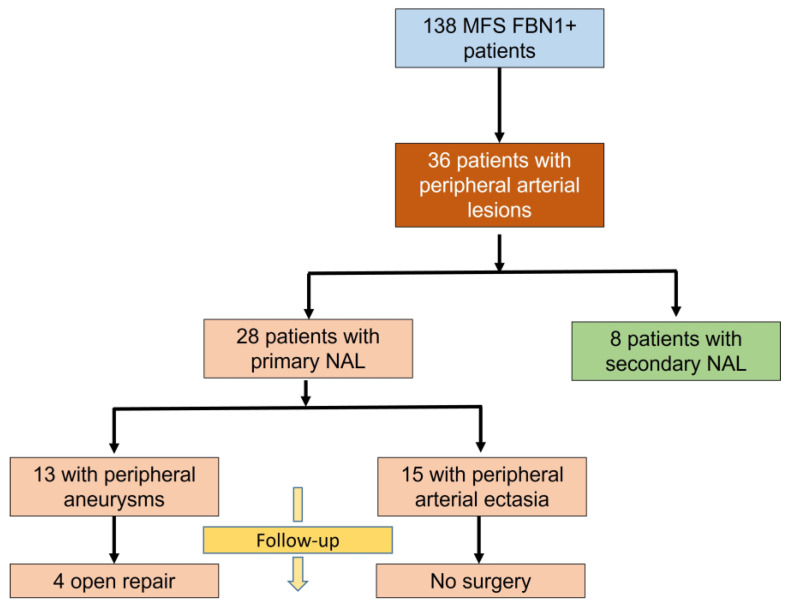
Study flowchart of the cohort and outcomes of patients presenting with primary non-aortic lesions (NAL).

**Figure A1:**
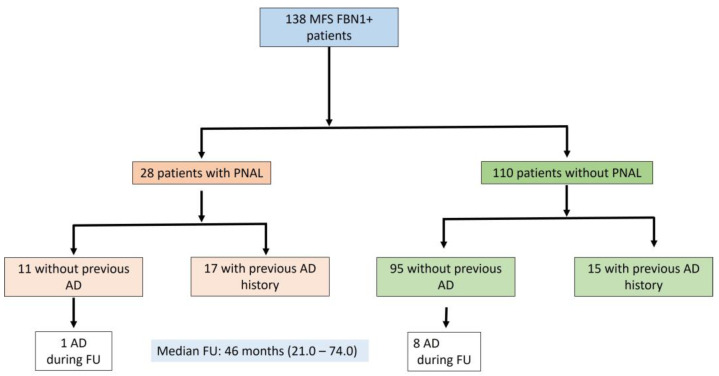
Study flowchart of the cohort, stratified by history and outcomes during follow-up of aortic dissection (AD). PNAL: primary non-aortic lesion.

In total, 138 patients (49.3% men) with FBN1 pathogenic variant were included. The cohort flowchart is shown in Figure 1. At baseline (i.e., at the date of the CTA allowing PNAL assessment), 48.5% (67/138) of patients had a history of AD (n = 32) and/or cardiac surgery (n = 62). In the 71 remaining patients, a CTA was performed to estimate evolutive ascending aorta enlargement in comparison to DUS during clinical FU in their MFS clinic. In this latter group, 29 (40.8%) patients underwent aortic event (7 AD and 1 death) or cardiac surgery (n = 21) during FU. At baseline, PNAL was found in 28 patients (20.3%) and secondary NAL in 19 patients (13.7%); this corresponds to a total of 36 patients presenting with NAL (26%). In total, 17 PNAL (60.7%) and 15 non-PNAL (13.6%) patients presented with history of AD (Figure A1, Appendix A). The median clinical follow-up of the cohort was 46.0 months (range 21–74, IQR: 53) with clinical follow-up durations of respectively 53.0 (28.5–73.8) vs 45.0 (16.0–74.5, *p* = 0.42) months in patients presenting with or without PNAL at baseline.

### 3.2. PNAL Population

A total of 27 aneurysms and 41 ectasias were found in the 28 PNAL patients. Of these, 9 patients presented with aneurysms only (6.5%), 15 patients with ectasias only (10.9%), and 4 patients with both aneurysms and ectasias (2.9%). Details of the cohort at baseline are presented in Table 1. Patients with PNAL were significantly older (44 years vs. 34 years, *p* = 0.001) and 24 (85%) had a history of cardiac surgery and/or AD at CTA. In the remaining four PNAL patients, three were treated for elective aortic surgery (mechanical Bentall procedure) and one presented with an acute type B dissection during FU. Among patients with AD at inclusion, 53.1% (17/32) had PNAL compared with 10.4% of patients without AD (11/106) (non-adjusted OR = 9.8, 95% CI: 3.9–24.9, *p* < 0.001). No differences were found in the presence of PNAL between patients with an FBN1 pathogenic variant resulting in haploinsufficiency and those with dominant negative mutations (*p* = 0.961).

### 3.3. Anatomical Distribution of PNAL (Table 2)

The most common locations of aneurysms were the subclavian artery (n = 8), the common iliac artery (n = 5), and the vertebral artery (n = 4), while ectasia was most reported in the iliofemoral (n = 29) and subclavian regions (n = 7). Ectasia was reported in a unique location in 5 patients and in multiple locations in 10. Patients with ectasia and those with aneurysms presented with similar characteristics and aortic features (Table 3).

### 3.4. Evolution of PNAL during Clinical Follow-Up

Among aneurysm lesions, four patients (4/13, 31%) with PNAL located in the renal, axillary, subclavian, and iliac arteries required surgery during follow-up, including one with a large renal aneurysm that had an imaging compatible with fibromuscular dysplasia. All procedures were open repairs and were performed in non-emergent settings. The maximal arterial diameters before open repair were 20 mm, 20 mm, 28 mm, and 55 mm for the renal, axillary, subclavian, and iliac aneurysms, respectively. Conversely, none of the patients with arterial ectasia required prophylactic surgery.

### 3.5. Association between PNAL and Aortic Dissection

**Figure 2:**
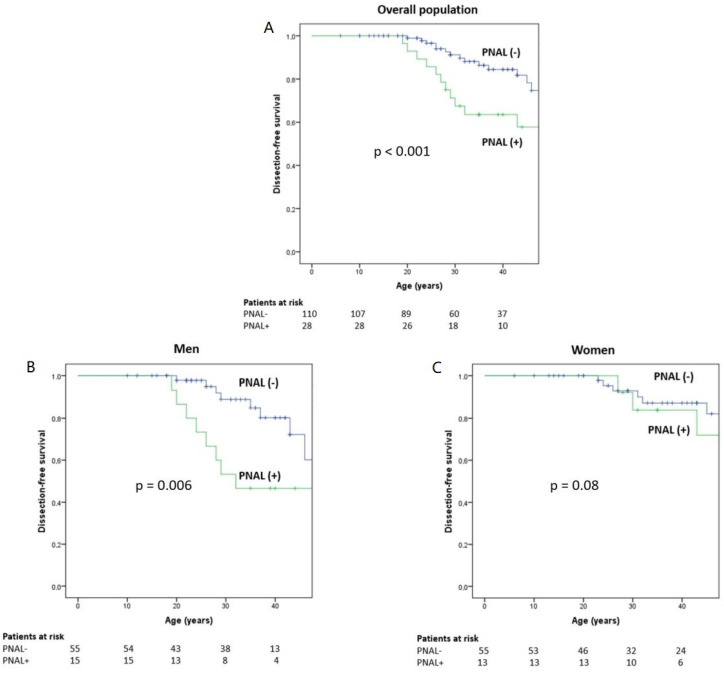
Kaplan–Meier estimates of freedom from aortic dissection for the overall population (**A**) and stratified by gender (**B**: men; **C**: women) for patients with primary non-aortic lesions (PNAL+) and without primary peripheral non-aortic lesions (PNAL-). Curves begin with the patients’ birth dates and end with the dates of the CTAs. Dissection may occur (step) or follow-up end (censoring mark).

No difference in AD prevalence was observed between patients with aneurysms and patients with ectasia. Patients with PNAL were older, but median age at AD was similar in both groups (31 vs. 32 years, *p* = 0.77). To compare the occurrence of AD in PNAL and non-PNAL patients during their lifespans, a survival analysis of events prior to baseline was performed using the Kaplan–Meier method. This analysis showed that AD occurred at an earlier age in the PNAL group (46.9 years, 95%CI: 39.6–54.1) compared with the non-PNAL group (59.6 years, 95%CI: 55.4–63.8, *p* < 0.001) (Figure 2A). This association was significant in men with PNAL (*p* = 0.006, Figure 2B) but not in women (*p* = 0.08, Figure 2C). Secondly, we sought to identify factors associated with the presence of PNAL using a multivariate analysis. Thus, previous AD (OR = 3.9, 95%CI: 1.3–12.1, *p* = 0.018), previous descending aortic surgery (OR = 10.3, 95%CI: 2.2–48.3, *p* = 0.003), and age (per 10 years OR = 1.6, 95%CI: 1.1–2.4, *p* = 0.008, Table 4) were significantly associated with the presence of PNAL. The Hosmer–Lemeshow goodness-of-fit test did not indicate a lack of fit in the model (*p* = 0.283).

## 4. Discussion

We report the occurrence of non-aortic lesions in patients with MFS related to FBN1 mutation in 20.3 % of our cohort. This value is in keeping with previous studies [7,8,9,10,13]. Moreover, it should be emphasized that we only report here primary lesions, i.e., not related to extension of an aortic dissection. This method differs from other reports [8] and is important to consider because failure to distinguish primary from secondary PALs (or aneurysms from ectasias) could lead to an overestimation of the prevalence of aneurysms, especially in the visceral and renal arteries. Such overestimation could also paradoxically lead to an underestimation of the percentage of non-aortic lesions as threatening lesions requiring surgery. For all these reasons, alongside other authors [8,9], we strongly advocate the need to define the standards of PNAL in Marfan patients, and their respective clinical prognosis.

Our results from a limited but very well-characterized population report distinct types of PNAL, namely ectasias (defined as enlarged arteries without loss of aortic wall parallelism) and aneurysms (defined as a loss of parallelism of the arterial walls associated with maximal diameters exceeding 150% of those of the adjacent segments). The most frequently reported PNAL were arterial ectasias in our series (60%). Multiple locations of PNAL were also frequently noticed with coexistence of aneurysms and ectasias in four patients. We observed that ectasias were present in multiple segments in one-third of patients (5/15), and that all surgeries required for PNAL patients during follow-up concerned only arterial aneurysms. This was probably partly the consequence of different maximal diameters between ectasias and aneurysms in our study.

The existence of PNAL clearly supports the idea that arterial disease in Marfan syndrome may be diffuse. Indeed, fibrillin-1 is a large, extracellular matrix (ECM) structural protein that polymerizes to form microfibrils. The latter play crucial roles in both the structural architecture of aortic and non-aortic arterial walls and mechano-transduction [14]. Such microfibrils link the vascular smooth muscular cells (vSMCs) to the elastin fibers in the aortic media (which includes almost 50 elastin layers) and also in the media of muscular arteries (as visceral arteries) mainly composed of vSMCs layers [14,15]. Distinguishing peripheral arterial ectasias from aneurysms in genetic diseases has been poorly discussed. Conversely, in the general population, vessel dilations (ectasias) are usually differentiated from focal vessel dilations (aneurysms) in accordance with their different natural histories, requiring different management in clinical practice although firm guidelines are absent. Whether or not the literature indicates that a pathophysiological continuum exists between peripheral arterial ectasias and aneurysms, it is to be noted that their association has been reported in up to 11% of patients presenting with lower limb degenerative aneurysms [16]. It is likely that haemodynamic conditions such as wall shear stress [17] or mechano-transduction [18] may depend on lesion morphology (i.e., differing between ectasias and aneurysms) and can impact the lesions’ outcomes. We recognize that our observation is based on a limited number of patients, as is often the case in rare diseases, and therefore requires confirmation by series from other centers. Until then, even though in our practice we do not consider non-aortic ectasias as aggressive lesions, we still recommend regular radiological follow-up in such settings. Indeed, (1) we could not assess the long-term outcomes of ectasias in our study due to limited (4 years) follow-up, (2) 4 out of 28 PNAL patients presented with both types of lesions, and (3) the MFS patients included in our cohort were young.

PNAL were mainly located in the iliac, subclavian, and femoral segments. This observation is consistent with other cohorts [10,13] reporting a majority of iliac and subclavian lesions in patients with Marfan syndrome. Other less frequently involved vessels include the intercostal [13], carotid [13], and vertebral arteries [8]. Cases of vascular involvement in exceptional locations, such as the renal [8], coeliac, superior mesenteric [13], and popliteal arteries, have also been reported [8]. In the present study, due to a lack of distal vascular assessment we were unable to estimate the prevalence of popliteal lesions. Further studies should aim to perform exhaustive evaluation of the arterial network using vascular ultrasound of the upper and lower limbs and cerebrovascular imaging to determine the accurate prevalence of primary aneurysm.

Interestingly, our results underscore the correlation of PNAL with aging and severe aortic disease. Indeed, Marfan patients presenting with PNAL were significantly older (44 vs. 34 years old in the non-PNAL group, *p* < 0.001), in keeping with the Spanish study findings [10]. Aortic dissection was also strongly associated with PNAL, independently of age. Age, history of AD, and history of descending aortic surgery were significant risk factors for PNAL with respective odd ratios of 1.6 (per ten years), 3.9, and 10.3. This is comparable to the observations reported by Mariucci et al. [13]. They showed that dilation of distal aortic segments and aortic branch vessels was more common in patients with previous aortic surgical replacement, suggesting a more aggressive and widespread vascular disease [13]. Aortic dissection-free survival in men in our study highlighted the severity of aortic disease in the presence of PNAL. The absence of statistical significance in women probably reflects the lack of power, as the IRAD registry [19] and more recent studies [20] report an incidence of acute thoracic AD twice as frequent in men. Similarly, there is a growing body of evidence that a male/female difference in disease severity is found in Marfan patients [10,12,21].

Several limitations can be underlined in our report. First, the estimation of the prevalence of PNALs may have been biased by the retrospective design of the study. This may have led to a non-exhaustive evaluation of the arterial network, as the lower limbs were not frequently screened in our series. Second, CTA screening was not systematic in clinical practice. This could have resulted in a selection bias for patients with more severe aortic disease (enriched in AD), which may have influenced the estimation of aortic event occurrence in the presence of an arterial aneurysm. Third, our datasets were limited to the separate study groups: this impaired a solid statistical-based analysis of the outcomes of aneurysms and ectasias during clinical follow-up, which remained limited in our study. This also hindered the statistical power of our retrospective analysis by including potential confounding factors in our multivariate analysis. Finally, we were unable to date the occurrence of PNAL and to monitor the PNAL growth rate during follow-up.

## 5. Conclusions

PNAL appeared to be present in 20% of the CTAs of our FBN1-positive population, similar to the reported rate in other studies. Surgery was required in 30.7% of patients with non-aortic aneurysms within a relatively short follow-up period. A gray area remains regarding whether peripheral arterial ectasias and aneurysms represent a continuum or are different diseases with different outcomes. Therefore, we suggest that CTA screening for non-aortic dilation should be performed in patients with MFS related to FBN1-mutation, especially in those with a history of AD, considering the benefits of arterial follow-up. To define the prognostic value of PNAL, the current findings require additional prospective and collaborative studies including larger cohorts, longer follow-up duration, and exhaustive evaluation of all non-aortic segments.

## Figures and Tables

**Table 1:** Patients’ characteristics at baseline for those presenting with or without primary non-aortic lesions (PNAL (+) or PNAL (-), respectively).

	PNAL (+) (n = 28)	PNAL (-) (n = 110)	Overall Population (n = 138)	*p*-Value
Age, median (IQR)	44.0 (27.0) *	34.0 (20)	36.5 (20.0)	<0.001
Men, n (%)	15 (53.6)	55 (50.0)	68 (49.3)	0.833
History of cardiac surgery, n (%)	23 (82.1)	70 (63.6)	93 (67.4)	0.073
Biological Bentall, n (%)	3 (4.3)	2 (8.7)	5 (5.4)	
Mechanical Bentall, n (%)	21 (30.0)	13 (56.5)	34 (36.6)	
Sus-coronary tube, n (%)	3 (4.3)	0 (0.0)	3 (3.2)	
Tirone David, n (%)	25 (35.7)	4 (17.4)	29 (31.2)	
Others, n (%)	18 (25.7)	4 (17.4)	22 (23.7)	
History of aortic dissection, n (%)	17 (60.7) *	15 (13.6)	32 (23.2)	<0.001
Type A, n (%)	8 (47.1)	8 (53.3)	16 (50.0)	
Type B, n (%)	5 (29.4)	5 (33.3)	10 (31.2)	
Age at aortic dissection, median (IQR)	31.0 (25)	32 (19)	32 (22)	0.770
History of descending aortic surgery, n (%)	12 (42.9) *	3 (2.7)	15 (10.9)	<0.001
Aortic open surgery, n (%)	9 (75.0)	1 (33.3)	10 (66.7)	0.242
TEVAR, n (%)	3 (25.0)	2 (66.7)	5 (33.3)	

* *p* < 0.001 for comparison Chi-square or Fisher’s exact test. Values are shown as median (interquartile range). Percentages in italics are calculated in relation to the respective count; IQR: interquartile range; TEVAR: thoracic endovascular aortic repair.

**Table 2:** Distribution of the primary non-aortic lesions.

Territory	Artery	Aneurysm		Ectasia	
		n	Size	n	Size
Neck	Internal carotid	0	NA	1	6 (NA)
	Common carotid	1	19.0 (NA)	1	15.0 (NA)
	Vertebral	4	9.0 (1.0)	0	NA
Chest	Axillary	3	20.0 (4.0)	0	NA
	Subclavian	8	17.1 (5.5)	7	13.0 (1.8)
	Innominate	1	20.0 (NA)	0	NA
Abdomen/Pelvis	Celiac artery	3	12.0 (1)	2	12.0 (1)
	SMA	1	15.0 (NA)	1	11.6 (NA)
	Renal	1	20.0 (NA)	0	NA
	Common iliac	5	21.0 (2.0)	17	16.0 (4.0)
	Hypogastric	0	NA	1	14.6 (NA)
	Femoral	0	NA	12	12.7 (2.4)

Aneurysms were not related to a previous aortic dissection nor cannulation site. Size is presented as mm (median, IQR); NA: not applicable; SMA: superior mesenteric artery.

**Table 3:** Baseline characteristics of patients presenting with PNAL at inclusion.

	Aneurysm Group (n = 13)	Ectasia Group (n = 15)	*p*-Value
Age, median (IQR)	44.0 (19)	51 (28)	0.40
Men, n (%)	8 (61)	7 (54)	0.98
History of cardiac surgery, n (%) ^a^	11 (85)	12 (80)	1
History of aortic dissection, n (%)	9 (69)	9 (60)	0.70
Type A, n (%) ^b^	6 (46)	6 (40)	
Type B, n (%) ^b^	5 (38)	5 (33)	
History of descending aortic surgery, n (%)	8 (62)	6 (40)	0.33
Open surgery, n (%) ^c^	6 (46)	3 (20)	
Endovascular repair, n (%) ^c^	2 (15)	3 (20)	

^a^ percentages are calculated in relation to the “history of cardiac surgery” count. ^b^ type A and type B dissection totals exceed the “history of aortic dissection” count because some patients experienced unrelated type A and type B dissection events (n = 6). ^c^ percentages are calculated in relation to the “history of aortic surgery” count. IQR: interquartile range.

**Table 4:** Univariate and multivariate analysis of factors associated with PNAL at baseline.

Variable		*p*-Value	OR	CI 95%	
				Lower	Upper
Univariate analysis	Gender	0.398	1.622	0.529	4.979
	History of AD	0.026	8.492	1.298	55.554
	History of descending aortic surgery	0.002	13.528	2.617	69.933
	History of cardiac surgery	0.687	1.319	0.342	5.078
	Age at CTA	0.007	1.727	1.163	2.565
Multivariate analysis	History of AD	0.018	3.896	1.257	12.073
	History of descending aortic surgery	0.003	10.323	2.209	48.250
	Age at CTA	0.008	1.636	1.139	2.349

The age at CTA is stratified per 10 years; AD: aortic dissection; CTA: computed tomography angiography; OR: odds ratio; CI: confidence interval.

## Data Availability

The datasets used and/or analyzed during the current study are available from the corresponding author on reasonable request. The data are not publicly available due to national regulation regarding privacy and ethics (Commission Nationale de l’Informatique et des Libertés, MR-004).
